# Annotations

**Published:** 1893-05-13

**Authors:** 


### Annotations.

**Milk in Bright's Disease.**

For several years past the writer has had doubts about the value of the milk treatment in cases of chronic albuminuria. Indeed, he has practically abandoned that particular diet as being productive of more harm than good. About a year ago a patient who was supposed to be suffering from Bright's disease had been so reduced by a milk diet that he declared he would rather die of the disease than be cured at the expense of prolonged starvation. Whether there had ever been any albuminuria it is impossible to say. But it is quite certain there has been none for the last twelve months, notwithstanding a full meat diet during the whole of that time. The recovery of the general health has been complete. The *Practitioner* for May quotes with approval Dr. Hale White's paper, read before the Medical and Chirurgical Society, in which he protests emphatically and with obvious justice against the exclusive or general use of a milk diet in Bright's disease. It is manifest that the milk diet is a purely theoretical method of treatment. The thesis might be thus expressed: There is albumin in the urine, where there ought to be none; stop the administration of albuminous foods and there will be none. That is the theory, but the practice has declined to conform itself thereto. An exclusively milk diet makes little difference in the quantity of albumin as a rule, but it often makes an extraordinary and fatal difference in the patient's strength, besides making meal-times a weariness to the flesh and spirit. It cannot be too often repeated that clinical experience is the judge and jury, whilst theoretical conclusions are only the witnesses. Let experimentation continue by all means, and let us leave nothing undone in order to establish a rational basis of theory. But when all is said, let us be thankful that the bedside offers us a final proof and test which can never make a mistake.

**Medical Practice in Australia.**

A few days ago an Australian medical man, who has something of a name in this country, called upon the present writer to ask his advice about settling in London. He has been driven out of the Australian colonies by the prevailing financial depression, and no doubt he is but a sample of many more colonial doctors, who will be speedily rushing to our shores to swell the already crowded ranks of the profession at home. On the subject of Australian practice generally Dr. Ludwig Bruck publishes a reprint from the *Australasian Medical Gazette* for March, 1893, which contains facts of the most startling and painful character. Among other things, Dr. Bruck says: "A large proportion of the population belong to friendly societies, the members of which include many well-to-do people, such as shopkeepers, manufacturers, members of Parliament, officials, and others. . . . For example, an important suburb of Sydney, on the western shores of Darling Harbour, with a population of 24,000, has two-thirds of the total population belonging to friendly societies. The Friendly Societies' Dispensary in that suburb alone has over 2,300 members, consisting, with their wives and children, of fully 10,000 persons. Those 10,000 are attended by two medical men only, who between them are paid no more than £600, or 1s. 2½d. for each person per annum. For this magnificent sum they have to attend, not only in sickness and accident, but also to perform minor operations, administer chloroform, and extract teeth. Another lodge of about 500 members (of course with their families) pays 15s. per member a year to its medical officer. This gentleman is not allowed even an extra fee for midwifery, but is obliged to attend all midwifery cases free of charge." For these fees, medicines, of course, have also to be provided. We reveal no secret when we affirm that medical practice of this kind must be of the most perfunctory, dishonest, and worthless character. The medicines, or substances called "medicines," must be of the poorest and most useless description. We publish these facts for two reasons: first, to prevent self-respecting medical men from going to Australia at the present time; and, secondly, to protest against exactly similar things which are now prevailing extensively in our own country. There are a class of men who destroy the reputation of medicine as an honest and trustworthy art, and who make it impossible for honest doctors to live in many parts of our larger and smaller towns. Now how is this condition of things brought about? By professional disloyalty and the absence of all business-like combination among the members of the profession itself. As the country grows richer the medical profession grows poorer. We have ourselves principally to thank for all this, and to that extent we are rightly served. But is it not time to put an end to it all, and by intelligent combination to make ourselves trustworthy and self-respecting men once more?

**Physiological Pessimism.**

The other day a doctor and a stockbroker, old friends, met, after an interval, and compared notes of their recent experiences of life. The doctor expressed the conviction that "life was not worth living." The stockbroker agreed with him to this extent, that life "would not be worth living except for the hope and conviction of a better life after death." The doctor affirmed that he did not believe in a life after death, and that life as it is, and as we know it, is not worth living. Such a meeting and conversation has not perhaps any very profound significance. The doctor was no doubt "hipped" for the moment, and the stockbroker was, perhaps, suffering from the general commercial depression and the absence of liveliness in the money market. But what strikes one as peculiar, by way of contrast, is that if one were to ask any ordinary farm labourer in the Northern counties or Scotland whether or not he considered life worth living, the man would stare, and wonder whether his questioner was sane. The farm labourer lacks every pleasure, every refinement, every superfluity, yet he is, on the whole, a bluff, hearty fellow, and well content with things as they are so long as he is in regular work and wages. What is the explanation of the mystery? Is it mental? Is it moral? Or is it physiological? It certainly is not mental. The doctor who "did not believe in a future life" was not expressing a disciplined and reasoned conviction, but the humour of a passing mood. The true explanation is physiological. The doctor and the stockbroker are town men. Their blood is imperfectly oxidised; their muscles, including the chief muscle, the heart, lack tone; their nervous systems generate no superfluous energy, no energy over and above the general needs, which may assume the character of delight in life. Their physical life is at so poor an ebb that it gives them more weariness than pleasure; and as the physical life, for most men, constitutes at least seven-tenths of the whole life, the balance of satisfaction very frequently dips the wrong way, and life is declared to be not worth living. But with the farm labourer the blood is splendidly oxidised. Every muscle, including the heart, is as strong and elastic as full vitality can make it. The brain is therefore thoroughly well nourished, sleep is sound, and the overplus of physiological energy takes the character of a slow and stolid but generally well-pleased content. To the farm labourer, deprived of everything but health, life is something to be cherished and prized. By the prosperous man of the middle class, who possesses everything but health, life is said to be not worth living. This is a problem which demands the attention of both priest and physiologist—of the sceptic and the man of faith.

